# Plasma-Derived Extracellular Vesicles as Potential Biomarkers in Heart Transplant Patient with Chronic Chagas Disease

**DOI:** 10.3201/eid2608.191042

**Published:** 2020-08

**Authors:** Nuria Cortes-Serra, Maria Tays Mendes, Clara Mazagatos, Joan Segui-Barber, Cameron C. Ellis, Cristina Ballart, Ana Garcia-Alvarez, Montserrat Gállego, Joaquim Gascon, Igor C. Almeida, María Jesús Pinazo, Carmen Fernandez-Becerra

**Affiliations:** Barcelona Institute for Global Health (ISGlobal), Universitat de Barcelona, Barcelona, Spain (N. Cortes-Serra, C. Mazagatos, J. Segui-Barber, C. Ballart, M. Gállego, J. Gascon, M.J. Pinazo, C. Fernandez-Becerra);; Border Biomedical Research Center, The University of Texas at El Paso, El Paso, Texas USA (M.T. Mendes, C.C. Ellis, I.C. Almeida);; Secció de Parasitologia, Departament de Biologia, Sanitat i Medi Ambient, Facultat de Farmàcia i Ciències de l’Alimentació, Universitat de Barcelona, Barcelona, Spain (C. Ballart, M. Gállego);; Arrhythmias Unit, Hospital Clinic, University of Barcelona, Barcelona, Spain (A. Garcia-Alvarez);; Institut d’Investigació en Ciències de la Salut Germans Trias i Pujol, Badalona, Spain (C. Fernandez-Becerra)

**Keywords:** Plasma, extracellular vesicles, biomarkers, Chagas disease, heart transplantation, proteomic analysis, Trypanosoma cruzi, parasites, Spain, Bolivia, tropical diseases

## Abstract

Chagas disease is emerging in countries to which it is not endemic. Biomarkers for earlier therapeutic response assessment in patients with chronic Chagas disease are needed. We profiled plasma-derived extracellular vesicles from a heart transplant patient with chronic Chagas disease and showed the potential of this approach for discovering such biomarkers.

Chagas disease, caused by *Trypanosoma cruzi* parasite, is one of the most prevalent parasitic infections in Latin America and is responsible for millions of clinical cases. However, mainly because of migratory movements, the epidemiology of Chagas disease has changed in recent decades; cases have increased substantially in North America, Europe, and Asia, where it is not endemic (*1*). Thus, raising awareness of this debilitating or deadly neglected tropical disease and promoting the creation of global strategies for its accurate diagnosis, treatment, and control are of paramount importance.

Detection of *T. cruzi*–specific antibodies in serologic assays is the current standard technique for diagnosing chronic Chagas disease. However, this so-called conventional serology is not a valid indicator of chemotherapeutic outcomes because most patients remain seropositive for 10–20 years after treatment (*2*). Therefore, validated biomarkers are lacking for early assessment of therapeutic responses for testing current and new drugs or treatment regimens.

Extracellular vesicles (EVs) are cell-derived membranous nanoparticles present in most biologic fluids. Biofluid-derived EVs are minimally invasive molecular tools for diagnosing and screening diseases (*3*). They can be released by various mammalian cells and pathogens, and their use as predictive biomarkers for disease progression and treatment outcomes has been reported for different pathologic conditions, including parasitic diseases (*3*,*4*).

## The Study

The Ethical Committee of Clinical Research of Hospital Clinic (Barcelona, Spain; reference no. Reg. HCB/2015/0616) approved this project. The patient provided written informed consent before sample collection.

In 2009, a 51-year-old patient from Bolivia with a history of chronic Chagas disease, exhibiting severe organ involvement (chronic cardiomyopathy Kuschnir III and megacolon and megaesophagus degree IV) (*5*), was admitted to the International Health Department (Hospital Clinic, Barcelona). Serologic diagnosis for chronic Chagas disease was performed using 2 ELISA kits (Ortho-Clinical Diagnostics, https://www.orthoclinicaldiagnostics.com) and BioELISA Chagas (Biokit, https://www.biokit.com). Together with clinical management of dysphagia and constipation, a pacemaker in the context of third-degree atrioventricular block was implanted. In July 2015, an echocardiogram revealed iterative cardiac failure and severe ventricular dysfunction (ejection fraction 15%–20%). On November 28, 2015, the patient underwent heart transplantation without incident, and results of follow-up endomyocardial biopsies showed no early signs of transplant rejection.

After transplantation and in the context of immunosuppression therapy (Table 1), quantitative PCR (qPCR) was performed weekly to detect *T. cruzi* in the blood (*Tc*-qPCR) (*7*). First benznidazole treatment was started when several consecutive and positive *Tc*-qPCRs confirmed Chagas disease reactivation. Three weeks after benznidazole treatment, the *Tc-*qPCR became negative. After completion of 80% of the treatment, bronchopulmonary aspergillosis developed, and the benznidazole course was interrupted. The *Tc-*qPCR became positive and a second benznidazole course was initiated; this time the patient completed the initial prescribed dose without evidence of therapeutic failure based on *Tc-*qPCR results. Plasma samples for purification and characterization of EVs were collected before the first benznidazole treatment and just after the second course (Table 1). Unexpectedly, the patient died in August 2016 because of a late organ rejection. Therefore, samples at 6 and 12 months posttreatment, already included in the approved protocol, were not collected.

**Table 1:** Timeline of heart transplant patient with chronic Chagas disease from initial diagnosis to last follow-up and death*

Date	Infection	Observation, treatment, outcome
2015 Aug	Cytomegalovirus, detected by serology	Diagnosed only by positive IgG serology, no active infection (no positive IgM serology). No treatment.
2015 Aug	Toxoplasmosis, detected by serology	Diagnosed only by positive IgG serology, no active infection (no positive IgM serology). No treatment.
2015 Nov		Heart transplantation on Nov. 28. Patient started with immunosuppressive therapy (tacrolimus, azathioprine, prednisone) until the end of follow-up.
2016 Jan	Chagas disease reactivation, detection by qPCR	Pretreatment sample collected on Jan 28. Patient started BZN treatment (2.5 mg/kg, twice a day, 60 d) on Feb 3.
2016 Mar	Bronchopulmonary aspergillosis, detected by serology and CT	BZN course interrupted on Mar 21. Completed 80% of the prescribed treatment.
2016 Mar	Bronchopulmonary aspergillosis	Aspergillosis treatment started on Mar 22. Initially with voriconazole and amphotericin B liposomal. Treatment was changed to posaconazole until the end of the follow-up.†
2016 Apr	Chagas disease reactivation, detected by qPCR	On Apr 14, patient started second round of BZN treatment until May 5, completing 100% of the prescribed treatment.
2016 May		Posttreatment sample collected on May 11.
2016 Aug		Late organ rejection. Patient died.

*BZN, benznidazole; CT, computed tomographic scan; qPCR, quantitative PCR. †Parasite clearance could be related to the prolonged used of posaconazole, as previously reported (*6*), and/or the combined use of posaconazole and benznidazole because a second round of the latter was started in April 2016.

**Figure 1:**
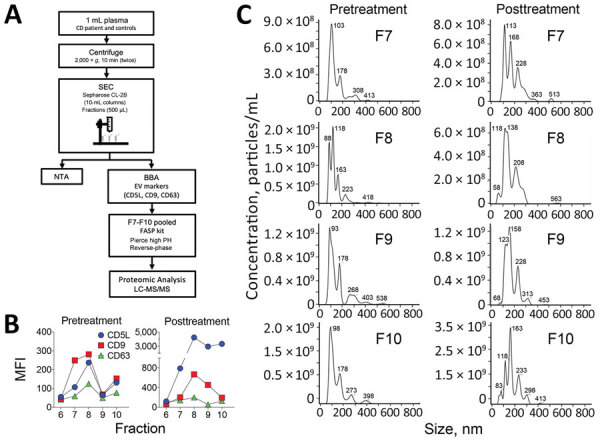
Isolation and characterization of plasma-derived EVs. A) Schematic diagram of the isolation and characterization of EVs derived from plasma samples. The details of each step are explained in The Study section. B) EVs were characterized by BBA using the classical EV markers CD5L, CD9, and CD63. C) NTA of SEC fractions F7–10. BBA, bead-based assay; EV, extracellular vesicle; LC-MS/MS, liquid chromatography–tandem mass spectrometry; MFI, median fluorescence intensity; NTA, nanoparticle tracking analysis; SEC, size-exclusion chromatography.

To determine whether circulating EVs from this patient could have been used as predictive biomarkers to evaluate therapeutic response and disease outcome in the Chagas disease context, we collected pretreatment and posttreatment plasma samples, and EVs were enriched by size-exclusion chromatography (SEC) and characterized as described (*8*) (Figure 1, panel A). As negative controls, plasma samples from 2 healthy donors were also subjected to SEC. We characterized eluting EVs by bead-based assay and Nanoparticle Tracking Analysis (Figure 1, panels B, C). We pooled aliquots (100 μL) from SEC fractions 7–10 and determined protein composition using 2D-liquid chromatography–tandem mass spectrometry (2D-LC-MS/MS). In brief, samples were digested with trypsin and resulting peptides were resolved by high-pH reversed-phase peptide fractionation (*9*), followed by C18 reversed-phase nanoflow ultrahigh-performance liquid chromatography coupled to a Q Exactive Plus Hybrid Quadrupole-Orbitrap Mass Spectrometer (QE Plus MS; Thermo Fisher Scientific, https://www.thermofisher.com), as described (*10*). Raw 2D-LC-MS/MS data were analyzed using Proteome Discoverer version 2.1.1.21 software (Thermo Fisher Scientific), followed by Scaffold perSPECtives version 4.8.7 (Proteome Software; http://www.proteomesoftware.com). A protein database with combined human, *T. cruzi*, and potential contaminants was generated from UniProt (https://www.uniprot.org). Using a false-discovery rate <1% and 1 unique peptide per protein, we identified 12 *T. cruzi* proteins and 338 human proteins (Appendix). However, when we applied the more stringent criterion of >2 unique peptides per protein, we detected only 1 *T. cruzi* protein (i.e., pyruvate phosphate dikinase [PPDK]), and 288 human proteins, of which we identified 19 only in pretreatment samples (Table 2). PPDK has been identified by proteomic analysis of *T. cruzi* total secretome and EVs (*10*–*12*). This protein plays a central role in the metabolism of *T. cruzi* glycosomes and has been shown to be upregulated when trypomastigote forms are incubated with the extracellular matrix, an obligatory step before host-cell invasion and differentiation of trypomastigote into amastigote forms (*13*). The specific role of PPDK in EVs secreted by this patient remains to be determined.

**Table 2:** *Trypanosoma cruzi* and human proteins identified in plasma-derived EVs from a heart transplant patient with chronic Chagas disease before benznidazole chemotherapy but absent after treatment and in healthy donors

Protein name†	UniProt accession no.	Unique peptides			
		ChD Pre-BZN‡	ChD Post-BZN	Healthy 1	Healthy 2
*T. cruzi*					
Pyruvate. phosphate dikinase OS = Trypanosoma cruzi marinkellei GN = MOQ_000480 PE = 3 SV = 1	K2MVM1_TRYCR	2 (0.96)	0	0	0
*Homo sapiens*					
Collagen α-1(VI) chain OS = Homo sapiens GN = COL6A1 PE = 1 SV = 3	CO6A1_HUMAN	3 (1.44)	0	0	0
Group of Angiopoietin-related protein 6 OS = Homo sapiens GN = ANGPTL6 PE = 1 SV = 1+1	ANGL6_HUMAN (+1)	3 (1.44)	0	0	0
sp|PPIA_HUMAN|	sp|PPIA_HUMAN|	3 (1.44)	0	0	0
Mannan binding lectin serine protease 2 OS = Homo sapiens GN = MASP2 PE = 1 SV = 4	MASP2_HUMAN	2 (1.92)	0	0	0
Myosin regulatory light chain 12B OS = Homo sapiens GN = MYL12B PE = 1 SV = 2	ML12B_HUMAN	2 (1.92)	0	0	0
Collagen α-2(VI) chain OS = Homo sapiens GN = COL6A2 PE = 1 SV = 4	CO6A2_HUMAN	2 (1.44)	0	0	0
Collectin subfamily member 10 (C-type lectin). isoform CRA_a OS = Homo sapiens GN = COLEC10 PE = 4 SV = 1	tr|A0A024R9J3|A0A024R9J3_HUMAN	2 (1.44)	0	0	0
Group of Coagulation factor XIII A chain OS = Homo sapiens GN = F13A1 PE = 1 SV = 4+2	F13A_HUMAN (+2)	2 (1.44)	0	0	0
Tyrosine 3-monooxygenase/tryptophan 5-monooxygenase activation protein. eta polypeptide. isoform CRA_b OS = Homo sapiens GN = YWHAH PE = 3 SV = 1	tr|A0A024R1K7|A0A024R1K7_HUMAN	2 (1.44)	0	0	0
Fibrinogen-like protein 1 OS = Homo sapiens GN = FGL1 PE = 1 SV = 3	FGL1_HUMAN	2 (0.96)	0	0	0
Group of L-lactate dehydrogenase A chain OS = Homo sapiens GN = LDHA PE = 1 SV = 2+1	LDHA_HUMAN (+1)	2 (0.96)	0	0	0
Group of Laminin subunit α-2 OS = Homo sapiens GN = LAMA2 PE = 1 SV = 1+1	A0A087WX80_HUMAN (+1)	2 (0.96)	0	0	0
Group of MHC class I antigen (Fragment) OS = Homo sapiens GN = HLA-A PE = 3 SV = 1+3	tr|D2KZ27|D2KZ27_ HUMAN (+3)	2 (0.96)	0	0	0
Group of Serum amyloid A protein OS = Homo sapiens GN = SAA1 PE = 1 SV = 1+2	E9PQD6_HUMAN (+2)	2 (0.96)	0	0	0
Group of Transforming growth factor β-induced 68kDa isoform 2 (Fragment) OS = Homo sapiens GN = TGFBI PE = 2 SV = 1+1	tr|A0A0S2Z4K6|A0A0S2Z4K6_HUMAN (+1)	2 (0.96)	0	0	0
Heparan sulfate proteoglycan 2 (Perlecan). isoform CRA_b OS = Homo sapiens GN = HSPG2 PE = 4 SV = 1	tr|A0A024RAB6|A0A024RAB6_HUMAN	2 (0.96)	0	0	0
Neurogenic locus notch homologue protein 3 OS = Homo sapiens GN = NOTCH3 PE = 1 SV = 2	NOTC3_HUMAN	2 (0.96)	0	0	0
V1–16 protein (Fragment) OS = Homo sapiens GN = V1–16 PE = 4 SV = 1	tr|Q5NV81|Q5NV81_ HUMAN	2 (2.88)	0	0	0
Rheumatoid factor RF-ET6 (Fragment) OS = Homo sapiens PE = 2 SV = 1	tr|A2J1N5|A2J1N5_ HUMAN	2 (5.29)	0	0	0

*BZN, benznidazole; ChD, Chagas disease. †All proteins were identified by at least 2 unique peptides. A unique peptide is defined as a peptide found only in >1 protein members of the same protein family of a certain proteome. ‡Normalized total spectrum count values are indicated in parenthesis. Complete mass spectrometry proteomic data have been deposited to the ProteomeXchange Consortium (http://proteomecentral.proteomexchange.org) via the PRoteomics IDEntifications Database (PRIDE, https://www.ebi.ac.uk/pride/) partner repository, with the dataset identifier PXD014668 and 10.6019/PXD014668.

Among the 19 human proteins uniquely identified in EVs from the patient with chronic Chagas disease before treatment, the mannan binding lectin serine protease 2 (MASP2) is worth highlighting. A recent study with human samples showed that *MASP2* gene polymorphisms and MASP2 levels are associated with high risk for chronic Chagas disease cardiomyopathy (*14*). Furthermore, mannose-binding lectin, which activates complement on *T. cruzi* through MASP2, has been related to a decrease in blood and tissue parasite load and in myocarditis and cardiac fibrosis in experimental *T. cruzi* infection (*15*). In this study, mRNA levels of collagen-1 and -6 increased in the infected animals’ hearts (*15*). These results could support our findings because collagen α-1 is one of the proteins identified exclusively in EVs before patient treatment (Table 2).

**Figure 2:**
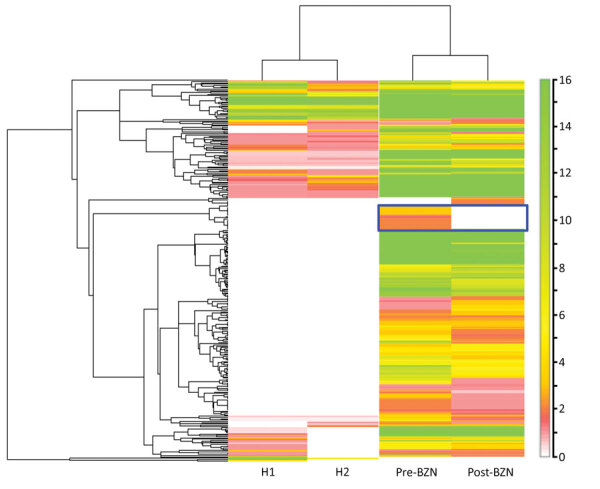
Human proteomic profile of plasma-derived EVs from a heart transplant patient with chronic Chagas disease before and after BZN treatment and from 2 healthy donors. Heatmap of the identified human EV-derived proteins. Heatmap was generated from proteomic data by the Scaffold perSPECtives software (Proteome Software, http://www.proteomesoftware.com). Hierarchical clustering was performed based on the normalized weighted spectrum count. Higher numbers of human proteins were found in EVs isolated from the patient, before and after benznidazole treatment, compared with EVs derived from the 2 healthy donors (H1, H2). Scale bar indicates intensity. BZN, benznidazole; EV, extracellular vesicle.

Another important observation is the identification of a higher number of human proteins in patient-derived EVs than in the 2 healthy donor–derived EV samples (Figure 2; Appendix). Of the total proteins identified, in which statistical analysis was feasible, 4 were significantly upregulated in patient-derived EVs before treatment, particularly for the proteins complement C1s subcomponent, isoform CRA_b, FLJ00385 protein, and cDNA FLJ75416 (Appendix). Complement C1s subcomponent recently was identified among the 6 upregulated EV biomarkers with potential for clinical applications in myocardial infarction (*3*).

## Conclusions

Proteins associated with EVs secreted by *T. cruzi* have been identified in the conditioned medium of different parasite stages (*11*–*13*) but not in biofluids from Chagas disease patients. We described the proteomic profiling of plasma-derived EVs purified directly from a heart transplant patient with chronic Chagas disease who exhibited reactivation after immunosuppression. We identified human and parasite proteins present or upregulated in plasma-derived EVs from a chronic Chagas disease patient before chemotherapy and that are absent or downregulated after treatment. We thus hypothesize that EV proteins released by the host or parasite during infection might be potential biomarker candidates for evaluating therapeutic response and disease outcome in chronic Chagas disease, independently of the immunologic status of patients.

However, our results should be interpreted with caution because they represent a single clinical case. Further research is needed to validate and provide stronger evidence that circulating EVs in patients with chronic Chagas disease can serve as biomarkers in disease progression and early assessment of therapeutic outcomes. Moreover, the future incorporation of such validated biomarkers in a point-of-care device could help in the detection of very low parasites in circulation, particularly when concentrations are below the PCR detection level (*2*).

Appendix Total human and parasite proteins identified in plasma-derived extracellular vesicles from 2 healthy donors and a patient with chronic Chagas disease before and after benznidazole treatment.
